# Supplementary material for: AffyMAPSDetector: a software tool to characterize Affymetrix GeneChip™ expression arrays with respect to SNPs
Source: BMC Bioinformatics. 2007 Jul 30;8:276. doi: 10.1186/1471-2105-8-276 (PMC1959249; doi:10.1186/1471-2105-8-276)
Supplement: Additional file 13 — AffyMAPSDetector v1 source code. AffyMAPSDetector source code. [file 1471-2105-8-276-S13.zip › AffyMAPSDetector_v1_src/doc/jwf/WizardListener.html]

WizardListener


|  |  |  |  |
| --- | --- | --- | --- |
| **Overview** \| **Package** \| **Class** \| **Use** \| **Tree** \| **Deprecated** \| **Index** \| **Help** | | |  |
| PREV CLASS   NEXT CLASS | **FRAMES**    **NO FRAMES**     **All Classes** |  |  |
| SUMMARY: NESTED \| FIELD \| CONSTR \| METHOD | DETAIL: FIELD \| CONSTR \| METHOD |  |  |


---


## jwf Interface WizardListener

**All Known Implementing Classes:**: FileLoaderWizardDialog, GeneAnnotLoaderWizard, WizardAdapter

---

public interface **WizardListener**

Implement this interface to be able to receive wizard related events.

**Author:**
:   Christopher Brind

---

|  |  |
| --- | --- |
| **Method Summary** | |
| `void` | `wizardCancelled(Wizard wizard)`             Called when the wizard is cancelled. |
| `void` | `wizardFinished(Wizard wizard)`             Called when the wizard finishes. |
| `void` | `wizardPanelChanged(Wizard wizard)`             Called when a new panel has been displayed in the wizard. |

**Method Detail**

### wizardFinished

```
public void wizardFinished(Wizard wizard)
```

:   Called when the wizard finishes.

    :   **Parameters:**: `wizard` - the wizard that finished.

---


### wizardCancelled

```
public void wizardCancelled(Wizard wizard)
```

:   Called when the wizard is cancelled.

    :   **Parameters:**: `wizard` - the wizard that was cancelled.

---


### wizardPanelChanged

```
public void wizardPanelChanged(Wizard wizard)
```

:   Called when a new panel has been displayed in the wizard.

    :   **Parameters:**: `wizard` - the wizard that was updated


---


|  |  |  |  |
| --- | --- | --- | --- |
| **Overview** \| **Package** \| **Class** \| **Use** \| **Tree** \| **Deprecated** \| **Index** \| **Help** | | |  |
| PREV CLASS   NEXT CLASS | **FRAMES**    **NO FRAMES**     **All Classes** |  |  |
| SUMMARY: NESTED \| FIELD \| CONSTR \| METHOD | DETAIL: FIELD \| CONSTR \| METHOD |  |  |


---
